# Space-time-coding digital metasurfaces

**DOI:** 10.1038/s41467-018-06802-0

**Published:** 2018-10-18

**Authors:** Lei Zhang, Xiao Qing Chen, Shuo Liu, Qian Zhang, Jie Zhao, Jun Yan Dai, Guo Dong Bai, Xiang Wan, Qiang Cheng, Giuseppe Castaldi, Vincenzo Galdi, Tie Jun Cui

**Affiliations:** 1 State Key Laboratory of Millimeter Waves, Southeast University, Nanjing, 210096 China; 2 Synergetic Innovation Center of Wireless Communication Technology, Southeast University, Nanjing, 210096 China; 3 Fields & Waves Lab, Department of Engineering, University of Sannio, I-82100 Benevento, Italy

## Abstract

The recently proposed digital coding metasurfaces make it possible to control electromagnetic (EM) waves in real time, and allow the implementation of many different functionalities in a programmable way. However, current configurations are only space-encoded, and do not exploit the temporal dimension. Here, we propose a general theory of space-time modulated digital coding metasurfaces to obtain simultaneous manipulations of EM waves in both space and frequency domains, i.e., to control the propagation direction and harmonic power distribution simultaneously. As proof-of-principle application examples, we consider harmonic beam steering, beam shaping, and scattering-signature control. For validation, we realize a prototype controlled by a field-programmable gate array, which implements the harmonic beam steering via an optimized space-time coding sequence. Numerical and experimental results, in good agreement, demonstrate good performance of the proposed approach, with potential applications to diverse fields such as wireless communications, cognitive radars, adaptive beamforming, holographic imaging.

## Abstract (web summary)

Current digital coding metasurfaces are only space-encoded. Here, the authors propose space-time modulated digital coding metasurfaces to obtain simultaneous manipulations of electromagnetic waves and present harmonic beam steering, beam shaping, and scattering-signature control as application examples.

## Introduction

During the past decades, electromagnetic (EM) metamaterials have experienced exponential developments due to their unique, finely tailorable properties that are not necessarily available in nature. Metamaterials are artificially engineered structures which have been widely used to manipulate EM waves in unconventional ways, leading to many exciting phenomena and novel devices^1^. Metasurfaces, as the two-dimensional (2D) equivalence of metamaterials, are attracting a steadily growing interest from researchers in both science and engineering communities, owing to their capability to provide abrupt phase shift, amplitude modulation (AM), and polarization conversion of EM waves^2–4^. Compared with three-dimensional (3D) bulk metamaterials, metasurfaces exhibit a negligible electrical thickness, thereby providing much better integrability and lower insertion losses. In 2011, Yu et al. put forward the idea of generalized Snell’s laws of reflection and refraction^2^, by designing a metasurface capable of impressing an abrupt phase shift, resulting in phase discontinuities that could be used to control (e.g., steer and focus) the light wavefronts. Since then, metasurfaces have experienced a fast-paced development, leading to many interesting devices capable of manipulating microwaves, terahertz waves, and visible light^5–10^. However, metasurfaces governed by the generalized Snell’s laws exhibit only space-gradient phase discontinuities on the interfaces, and are inherently constrained by Lorentz reciprocity and time-reversal symmetry. In 2015, Hadad et al. proposed the concept of space-time gradient metasurfaces^11,12^, by applying time modulation to the electronic properties of the surface impedance, thereby attaining space-time modulation of the EM waves and breaking the time-reversal symmetry. In the same year, Shaltout et al. proposed time-varying metasurfaces by introducing a time-gradient phase discontinuity^13^, which can break Lorentz reciprocity and bring about a new degree of freedom in controlling EM waves. The reader is also referred to refs. ^14–17^ for more recent examples of applications and modeling of space-time-modulated metasurfaces. It is worth stressing that most of the above studies deal with theoretical and numerical investigations, whereas experimental realizations remain quite limited.

On the other hand, digital coding and programmable metasurfaces have rapidly evolved since they were initially proposed in 2014^18–33^. These structures were originally put forward to digitally control EM waves, by designing two distinct coding elements with opposite reflection phases (e.g., 0° and 180°) as the digital bits “0” and “1” (binary case)^18^. This concept can be extended from binary (1-bit) to multi-bit configurations. For example, a 2-bit coding metasurface is constructed by a sequence of four coding elements “00”, “01”, “10”, and “11”, exhibiting 0°, 90°, 180°, and 270° phase responses, respectively. Digital coding metasurfaces could greatly simplify the design and optimization procedures since the phase of the reflection (or transmission) coefficient is digitally discretized in the unit-cell and system designs, thereby substantially reducing the parameter search space. By arranging the coding elements on a 2D plane with pre-designed coding sequences, such metasurfaces can be used to manipulate EM waves in a simple and effective way. It is worth noting that the application range of coding metasurfaces is not limited to microwave frequencies, but it has also been extended to the terahertz band^19–22,31^, as well as to acoustic scenarios^23^.

Remarkably, digital coding metasurfaces have built a bridge between the physical and digital worlds, making it possible to revisit metamaterials from the perspective of information science^29–31^. Most importantly, the digital description of coding metasurfaces is naturally suited to the integration with active elements, such as positive-intrinsic-negative (PIN) diodes, varactors, and micro-electro-mechanical systems (MEMS). Therefore, all coding elements of a digital coding metasurface can be independently controlled by a field-programmable gate array (FPGA). By changing the coding sequences stored in the FPGA, many different functionalities can be switched in real time, thereby leading to programmable metasurfaces. Digital coding and programmable metasurfaces have been employed successfully to generate vortex beams^32,33^, reprogrammable holograms^26^, reflect/transmit arrays^20–22,24,25^, diffuse scattering^19,27,28^, etc.

In the above described digital programmable architectures, however, only space coding has hitherto been considered, whereas the time dimension has not been exploited. In other words, the coding sequences are generally fixed in time, and are changed by the control system only to switch the functionalities whenever needed. As previously mentioned, time modulation of metasurfaces has been suggested in refs. ^11–13^, but these theoretical approaches were based on analog modulations, which are very difficult to realize in practice. As an alternative viable route, we introduce the concept of space-time-coding to extend the arsenal of metasurface-based wave manipulations.

Accordingly, in this paper, we consider space-time-coding digital metasurfaces based on the time modulation of the reflection coefficient. Specifically, a set of coding sequences are switched cyclically in a predesigned time period, leading to the desired harmonic scattered-power distributions in the frequency domain. To some extent, our approach combines the concept of digital coding metasurfaces with that of “phase-switched screens”^34^ and “time-modulated arrays”^35^. Firstly, we outline the fundamental theory of time-varying coding metasurfaces based on the Fourier transform method. Subsequently, we present several illustrative examples to demonstrate the potential of our approach in manipulating the spectral powers. The first example deals with attaining harmonic beam steering by exploiting a binary particle swarm optimization (BPSO) algorithm to design the space-time-coding sequences. The second and third examples are focused on beam steering and shaping at the central frequency, and the remaining examples are aimed at reducing the scattering by suitably redistributing the power across the frequency spectrum. Finally, an 8 × 8 space-time coding and programmable metasurface loaded with PIN diodes is fabricated and experimentally tested. The proposed approach is expected to broaden the applications of digital coding metasurfaces significantly, and promises important advantages in scenarios such as wireless communication^36,37^ and radar systems^38^.

## Results

### Theory

**Fig. 1:**
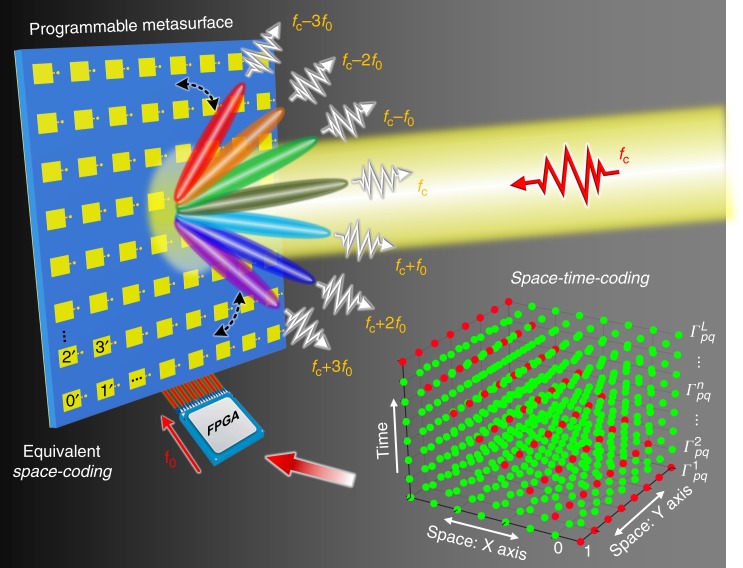
Conceptual illustration of a space-time-coding digital metasurface. The reflection coefficient of the metasurface elements is dynamically controlled with discrete phase or amplitude states by applying control voltages to PIN diodes. In the illustrated example, the reflection phase of each coding element is periodically switched according to the digital “0/1” space-time-coding matrix, which can result in an equivalent multi-bit space-coding, such as “0′”, “1′”, “2′” and “3′” (equivalent 2-bit case). This coding strategy enables precise control of EM waves in both space and frequency domains

We consider a space-time-coding digital metasurface that contains a 2D array of *M* × *N* elements loaded with PIN-diodes, as shown in Fig. 1. By applying a control voltage to a PIN diode, the reflection coefficient of the element can be dynamically controlled with discrete phase or amplitude states. For the 1-bit case, the reflection phase or amplitude of each coding element can be periodically switched according to the digital “0/1” space-time-coding matrix in the bottom-right corner of Fig. 1, in which the red and green dots represent the “1” and “0” digits, respectively. The space-time-coding strategy enables simultaneous control of EM waves in both spatial propagation and harmonic power distribution.

By assuming the time-modulation speed much smaller than the EM-wave frequency, and based on physical-optics-type approximations, we can adiabatically extend the approximate modeling that was originally introduced for space-coding metasurfaces^18^. Accordingly, under normal incidence of plane waves with suppressed time-harmonic dependence exp(*j*2*πf*_c_*t*_c_), the time-domain far-field pattern scattered by the space-time-coding digital metasurface can be expressed approximately as

1 $$	f\left( {\theta ,\varphi ,t} \right) = \mathop {\sum}\limits_{q = 1}^N \mathop {\sum}\limits_{p = 1}^M E_{pq}\left( {\theta ,\varphi } \right)\Gamma _{pq}\left( t \right) \\ 	\exp \left\{ {j\frac{{2\pi }}{{\lambda _{\mathrm{c}}}}}  \\ 	{\left[ {\left( {p - 1} \right)d_x\sin \theta \cos \varphi + \left( {q - 1} \right)d_y\sin \theta \sin \varphi } \right]} \right\}$$

where *E*_*pq*_(*θ*,*φ*) is the far-field pattern pertaining to the (*p*, *q*)th coding element computed at the central frequency *f*_c_, *θ* and *φ* are the elevation and azimuth angles, respectively, *d*_*x*_ and *d*_*y*_ are the element periods along the *x* and *y* directions, respectively, and *λ*_c_ is the central operational wavelength. According to the time-switched array theory^34,35^, Γ_*pq*_(*t*) is the time-modulated reflection coefficient of the (*p*, *q*)th element, which is assumed as a periodic function of time and defined over one period as a linear combination of shifted pulse functions:

2 $$\Gamma _{pq}\left( t \right){\mathrm{ = }}\mathop {\sum}\limits_{n = 1}^L {\Gamma _{pq}^n} U_{pq}^n\left( t \right)\quad \left( 0\hskip 2pt < \hskip 2pt t \hskip 2pt < \hskip 2pt T_0 \right)$$

where *U*^*n*^_*pq*_(*t*) is a periodic pulse function with modulation period *T*_0_. In each period, we have

3 $$U_{pq}^n\left( t \right) = \left\{ \begin{array}{l}1,\left( {n - 1} \right)\tau \le t \le n\tau \\ \hskip -35pt 0,{\mathrm{otherwise}}\end{array} \right.$$

where *τ* = *T*_0_/*L* is the pulse width of *U*^*n*^_*pq*_(*t*), *L* is a positive integer representing the length of the time-coding sequence, and Γ^*n*^_*pq*_ = *A*^*n*^_*pq*_exp(*jφ*^*n*^_*pq*_) is the reflection coefficient of the (*p, q*)th coding element during the interval (*n* − 1)*τ* ≤ *t* ≤ *nτ* at the central frequency, with *A*^*n*^_*pq*_ and *φ*^*n*^_*pq*_ denoting the amplitude and phase, respectively.

Next, we decompose *U*^*n*^_*pq*_(*t*) into a Fourier series

4 $$U_{pq}^n\left( t \right) = \mathop {\sum}\limits_{m = - \infty }^\infty {c_{pq}^{mn}} \exp \left( {j2\pi mf_0t} \right)$$

where *f*_0_ = 1/*T*_0_, and the Fourier coefficients $c_{pq}^{mn}$ are given by

5 $$c_{pq}^{mn} = \frac{1}{{T_0}}{\int}_0^{T_0} {U_{pq}^n\left( t \right)\exp \left( { - j2\pi mf_0t} \right)} dt$$

Thus, the Fourier series coefficients $a_{pq}^{\mathrm{m}}$ of the periodic function $\Gamma _{pq}\left( t \right)$ can be represented as (see Supplementary Note 1 for detailed derivations)

6 $$\begin{array}{l}a_{pq}^m = \mathop {\sum}\limits_{n = 1}^L {\Gamma _{pq}^n} c_{pq}^{mn} = \mathop {\sum}\limits_{n = 1}^L {\frac{{\Gamma _{pq}^n}}{{T_0}}} {\int}_{\left( {n - 1} \right)\tau }^{n\tau } {e^{ - j2\pi mf_0t}}dt \\ = \mathop {\sum}\limits_{n = 1}^L {\frac{{\Gamma _{pq}^n}}{L}{\mathrm{sinc}}\left( {\frac{{\pi m}}{L}} \right){\mathrm{exp}}\left[ {\frac{{ - j\pi m\left( {2n - 1} \right)}}{L}} \right]} \end{array}$$

Finally, the far-field scattering pattern of the space-time-coding digital metasurface at the *m*th harmonic frequency *f*_c_ + *mf*_0_ is written as

7 $$\begin{array}{l}\hskip-76ptF_m\left( {\theta ,\varphi } \right) = \mathop {\sum}\limits_{q = 1}^N \mathop {\sum}\limits_{p = 1}^M E_{pq}\left( {\theta ,\varphi } \right)\\ \hskip-6pt {\mathrm{exp}}\left\{ {j\frac{{2\pi }}{{\lambda _{\mathrm{c}}}}\left[ {\left( {p - 1} \right)d_x{\mathrm{sin}}\;\theta\; {\mathrm{cos}}\;\varphi + \left( {q - 1} \right)d_y{\mathrm{sin}}\;\theta\; {\mathrm{sin}}\;\varphi } \right]} \right\} a_{pq}^m\end{array}$$

In the approximate modeling, the mutual coupling between coding elements is ignored. Hence, for an arbitrary 3D space-time-coding sequence (see Supplementary Note 2 for details), we can calculate the scattering pattern of the coding metasurface at any harmonic frequencies via Eq. (7). Throughout the paper, for simplicity, we assume isotropic coding elements (*E*_*pq*_ = 1). By controlling the time-coding sequences of the individual elements, a set of complex reflection coefficients $a_{pq}^m$ is synthesized to control their scattering properties. More specifically, via Eq. (6), we can synthesize the equivalent amplitude and phase excitations of all elements at a specific harmonic frequency, as shown in Supplementary Figure 1.

In this study, we assume that the reflection amplitude $A_{pq}^n$ of each element is uniform, whereas the phase $\varphi _{pq}^n$ is a periodic function of time, whose values are either 0° or 180°, according to the digits “0” and “1” in the coding sequence. Henceforth, we shall refer to this scheme as “phase modulation” (PM). The coding sequence in the space-time-coding digital metasurface can be arbitrary, and is represented by a 3D matrix with dimensions (*M, N, L*). For instance, Supplementary Figure 1a displays a random 3D space-time-coding matrix with dimensions (8, 8, 8), representing a coding metasurface composed of 8 × 8 elements, with an 8-interval periodic time modulation (see also Supplementary Figure 1f). All coding elements are periodically switched according to the 3D matrix shown in Supplementary Figure 1a. Each element has its own independent time-coding sequence, which results in different equivalent amplitudes and phases at the various harmonic frequencies. For instance, with reference to the +2nd harmonic frequency, the equivalent amplitude and phase distributions are shown in Supplementary Figures 1b, c, respectively. The corresponding 3D and 2D scattering patterns in the *uv*-plane (*u* = sin *θ* cos*φ*, *v* = sin *θ* sin *φ*) are shown in Supplementary Figures 1d, e, respectively. The reader is referred to Supplementary Figures 2 and 3 for additional results.

As a key aspect of the proposed approach, we highlight that, although the physical coding elements exhibit only binary reflection phases (0° or 180°), the equivalent excitation [via Eq. (6)] can attain almost 360° phase coverage by suitably designing the time-coding sequences, as shown in Supplementary Figures 1c. In this way, a single 1-bit (or 2-bit) programmable metasurface periodically time-switched can be used to effectively synthesize multi-bit programmable metasurfaces (see Supplementary Note 3), which can open up a plethora of interesting applications.

### Harmonic beam steering

**Fig. 2:**
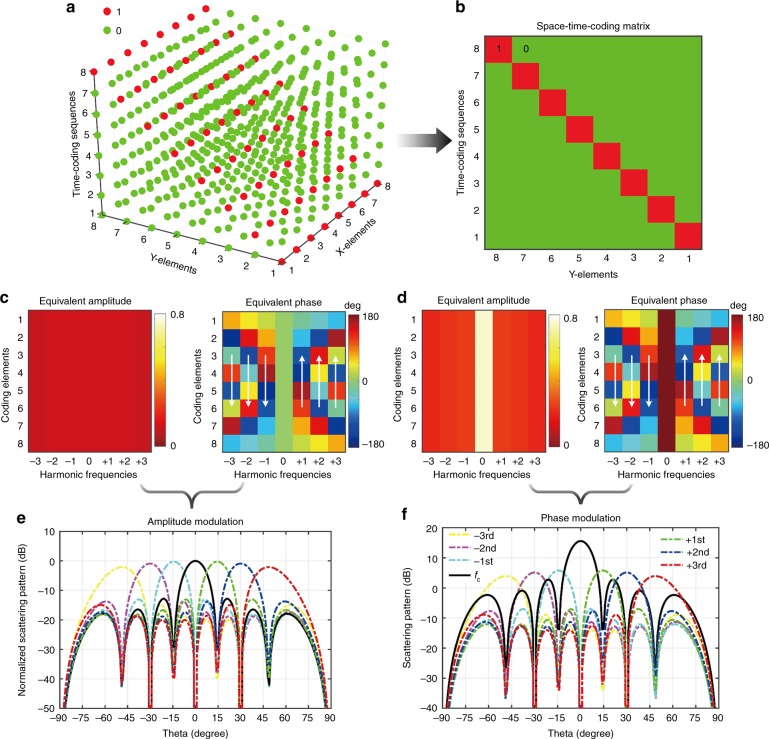
Harmonic beam steering. **a**, **b** 3D space-time-coding matrix and corresponding 2D coding matrix, respectively. The red and green dots represent “1” and “0” digits, respectively. **c**, **d** Equivalent amplitude and phase distributions pertaining to AM and PM schemes, respectively. The white arrows indicate the phase gradients that emerge at the various harmonic frequencies. **e**, **f** Corresponding 1D scattering pattern cuts (at *φ* = 90°) at different harmonic frequencies pertaining to AM and PM schemes, respectively

With the aid of the above theoretical analysis, we can successfully attain precise control of the scattering patterns at any harmonic frequencies. As a first illustrative example, we address the design of a space-time-coding matrix for harmonic beam steering in one plane. Traditional time-switched arrays considered in previous studies^39,40^ can realize harmonic beam steering by using time-gradient sequences, as illustrated in Fig. 2a, b. However, these methods are based on AM, in which only one element of the array scatters at a given time, thereby resulting in significant gain reductions. Conversely, if we use the same time sequences, but considering the proposed PM scheme instead, the scattered powers can be significantly enhanced. The equivalent amplitudes and phases of the coding elements under AM and PM are shown in Fig. 2c, d, respectively. We clearly observe that the equivalent amplitudes pertaining to AM are almost constant, and significantly smaller than the PM ones, which confirms our intuition above. Moreover, it can also be observed that some equivalent phase gradients emerge at positive and negative harmonic frequencies (see white arrows), which are instrumental to steer the harmonic beams. The harmonic beam steering can be better understood by introducing a time shift in the Fourier transform. The time-coding sequences of the Y-elements from 1st to 8th in Fig. 2b can be considered as the periodic function Γ_*pq*_(*t*) with a time shift *t*_*q*_, whence $\Gamma _{pq}(t - t_q)\mathop{\longleftrightarrow}\limits^{{FS}}a_{pq}^m\exp \left( { - j2\pi mf_0t_q} \right)$. Accordingly, the time shift brings an additional space phase shift $- 2\pi mf_0t_q$ with unchanged amplitudes at the *m*th harmonic frequency, which clearly explains the phase gradients in Fig. 2c, d (see Supplementary Note 4 for more details). Figure 2e shows the normalized scattering patterns pertaining to AM at different harmonic frequencies, while Fig. 2f shows the PM ones (normalized with respect to those in Fig. 2e). The scattering peaks pertaining to PM are 15.56, 5.78, 5.11, and 3.90 dB higher than the AM ones at *f*_c_, *f*_c_ ± *f*_0_, *f*_c_ ± 2*f*_0_, and *f*_c_ ± 3*f*_0_, respectively. However, the power levels in the PM case are markedly unbalanced, as the power at the central frequency is much larger than those at other harmonic frequencies.

**Fig. 3:**
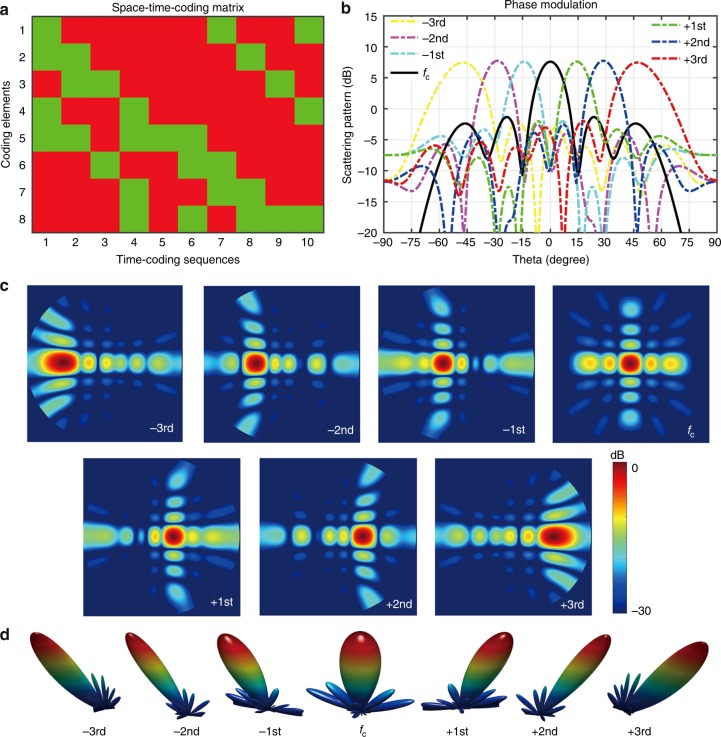
BPSO-optimized harmonic beam steering. **a** Optimized 2D space-time-coding matrix. **b**, **c**, **d** Corresponding 1D scattering-pattern cuts at *φ* = 90°, 2D, and 3D scattering patterns, respectively, at different harmonic frequencies

For better equalization of the power levels, we exploit a BPSO algorithm to optimize the time-coding sequence of each coding element (see Supplementary Note 5 for more details). As a result, we obtain an optimized 2D space-time coding matrix, as shown in Fig. 3a. The equivalent amplitude and phase of this coding matrix are shown in Supplementary Figures 5a and 5b, respectively (see Supplementary Note 6 for details). The corresponding harmonic scattering patterns are shown in Fig. 3b, from which we observe that the power levels at different harmonic frequencies are now uniform and about 7.6 dB higher than those pertaining to AM (cf. Fig. 2e). The 2D and 3D scattering patterns are displayed in Fig. 3c, d, respectively. It can be observed that the main beams at different harmonic frequencies point to different directions, thereby realizing the desired harmonic beam steering.

It is worth pointing out that the harmonic scattering patterns can also be calculated via the inverse fast Fourier transform (IFFT) technique^31^, which can greatly reduce the computational complexity of the optimization, especially for electrically large metasurfaces.

### Beam steering and shaping at the central frequency

In the above designed 1-bit space-time-coding metasurface, only the beams at harmonic frequencies are steered towards other directions, whereas the beam at the central frequency always points at broadside. This limitation essentially stems from the equivalent phase at the central frequency (see Fig. 2c, d and Supplementary Figure 4a). As anticipated, we could use physical coding elements with 2-bit phase responses to obtain 3-bit equivalent space coding at the central frequency. The space-time-coding strategy provides a new way of designing multi-bit programmable metasurface (even with arbitrary phases), which does not require a complicated layout and control system, and enables a more precise control of EM waves in space and frequency domains.

**Fig. 4:**
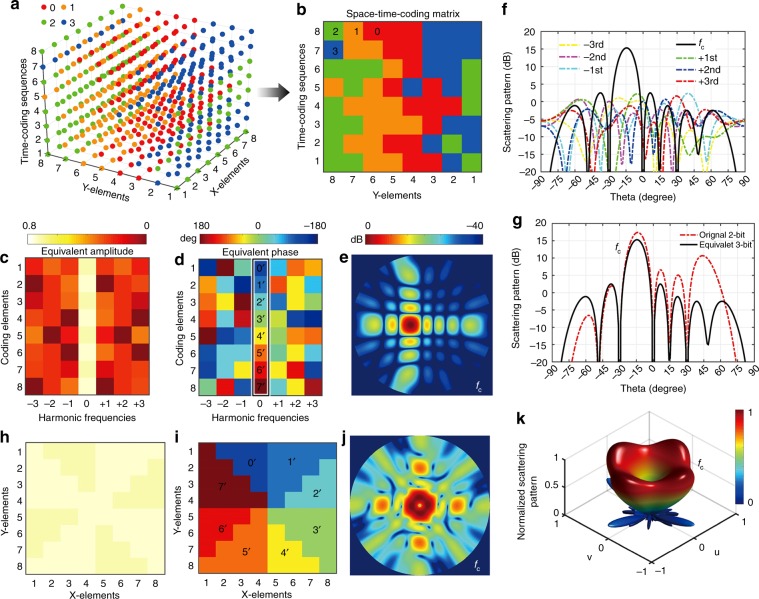
Beam steering and shaping at the central frequency. **a**, **b** 3D space-time-coding matrix and corresponding 2D coding matrix for beam steering, respectively, in which the red, yellow, green, and blue dots represent “0”, “1”, “2”, and “3” digits, respectively. **c**, **d** Equivalent amplitude and phase distributions at different harmonic frequencies, respectively. This space-time-coding matrix results in equivalent 3-bit coding responses “0”, “1′”, “2′”, “3′”, “4′”, “5′”, “6′”, and “7′” at the central frequency. **e** Corresponding 2D scattering pattern at the central frequency for beam steering. **f** Corresponding 1D scattering pattern cuts (at *φ* = 90°) at different harmonic frequencies. **g** Comparison of scattering patterns pertaining to the original 2-bit coding and equivalent 3-bit coding for realizing the beam steering at the central frequency. **h**, **i** Equivalent amplitude and phase distributions for vortex-beam generation at the central frequency, which exhibit an equivalent 3-bit coding spiral phase profile (OAM mode *l* = 1). **j**, **k** Corresponding 2D and 3D scattering patterns at the central frequency for beam shaping, respectively

More specifically, we consider a coding metasurface with 8 × 8 elements and time-coding sequences with a length of 8. First, we address the beam-steering design in one plane at the central frequency. Hence, we only need to study time-coding sequences of eight elements, and each column of elements has the same digital code. Figure 4a, b shows the 2-bit space-time-coding matrix, in which the red, yellow, green, and blue dots represent “0”, “1”, “2”, and “3” digits, representing 0°, 90°, 180°, and 270° phase responses, respectively (the criterion for selecting those eight sets of time-coding sequences is detailed in Supplementary Note 7). The equivalent amplitudes and phases of those eight coding elements at different harmonic frequencies are shown in Fig. 4c, d, respectively. We can clearly observe that the designed space-time-coding matrix effectively results in equivalent 3-bit responses, “0^′^” (−135°), “1^′^” (−90°), “2^′^” (−45°), “3^′^” (0°), “4^′^” (45°), “5^′^” (90°), “6^′^” (135°), and “7^′^” (180°), and an equivalent phase gradient at the central frequency. According to the generalized Snell’s law^2^, this phase discontinuity will steer a normally incident wave by an angle of 14.5°. The corresponding 2D scattering pattern at the central frequency and the scattering patterns at different harmonic frequencies are displayed in Fig. 4e, f. It can be seen that the main beam at the central frequency points at *θ* = −14.5°, and the scattered powers at the harmonic frequencies are much smaller, which provides good sideband suppression. Moreover, we compare the beam-steering performance of the original 2-bit coding “0-0-1-1-2-2-3-3” with the equivalent 3-bit coding “0^′^-1^′^-2^′^-3^′^-4^′^-5^′^-6^′^-7^′^”, as shown in Fig. 4g. It is observed that the former scheme could realize a steering angle of 14.5°, but accompanied by high sidelobes, while the latter scheme attains the steering with much lower sidelobes and only a little gain reduction attributable to the sideband power losses. Similar examples of equivalent 3-bit coding for large-angle beam steering are illustrated in Supplementary Figures 6c and 6d.

As a further illustrative example, we exploit the eight sets of time-coding sequences in Fig. 4b to generate a vortex beam carrying orbital-angular-momentum (OAM) at the central frequency. The coding metasurface is spatially divided into eight sectors with rotated distribution of time-coding sequences, and each sector has the same time-coding sequence. The final space-time-coding configuration is displayed in Supplementary Figure 7a, while the corresponding equivalent amplitudes and phases at the central frequency are shown in Fig. 4h, i, respectively. The equivalent 3-bit space-coding in Fig. 4i exhibits a spiral-like phase profile, and can generate a vortex beam with OAM mode *l* = 1^2,32^. The corresponding 2D and 3D scattering patterns at the central frequency are shown in Fig. 4j, k. Consistently with the characteristics of the desired vortex beam, a typical intensity profile with a hollow center can been clearly observed.

It is worth highlighting that the above examples, dealing with radiation scenarios of practical interest for high-performance antennas and wireless communication systems, could not be readily addressed via the simpler strategies (phase-switched screens or time-modulated arrays) in refs. ^34,35^.

### Scattering control for radar-cross-section reduction

In radar and stealth technologies, reducing the scattering from a target, i.e., reducing its radar cross section (RCS), represents a pivotal issue. Conventional radar absorbing materials can absorb the incident EM power, whereas low-scattering metasurfaces based on the principle of phase cancellation can redirect the incident wave to other directions^18,19,27,28,41^. The phase-switched screen proposed in ref. ^34^ exploits temporal-coding (via a simple time-switching sequence “01”) for redistributing the scattered power to odd harmonics. Against this background, our proposed space-time-coding digital metasurfaces can redistribute the scattered power in both space and frequency domains, leading to a mechanism for RCS reductions that completely differs from the conventional strategies.

**Fig. 5:**
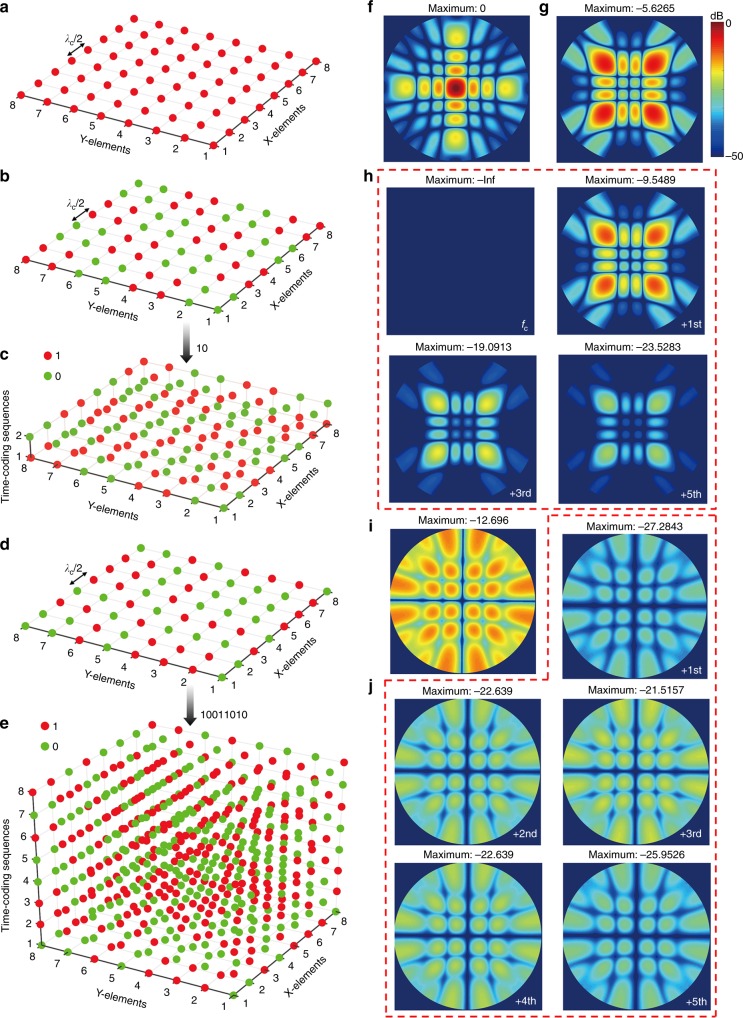
RCS reductions via space-time-coding metasurfaces. **a**, **b** Metasurfaces with uniform and chessboard-like space-coding distributions, respectively. **c** 3D space-time-coding matrix obtained by applying the time-coding sequence “10” to the chessboard-like space coding. **d** Metasurface with an optimized space-coding distribution. **e** 3D space-time-coding matrix obtained by applying the random time-coding sequence “10011010” to the space coding in panel (**d**). **f**, **g** 2D scattering patterns pertaining to the metasurfaces in panels (**a**, **b**), respectively. **h** 2D scattering patterns pertaining to the space-time-coding digital metasurface in panel (**c**) at different harmonic frequencies. Note that this time-coding results in the generation of odd harmonics only. **i** 2D scattering patterns of the metasurface in panel (**d**). **j** 2D scattering patterns pertaining to the space-time-coding digital metasurface in panel (**e**) at different harmonic frequencies

For example, Fig. 5a illustrates a metasurface featuring a uniform reflection-phase distribution, which essentially behaves like a metallic plate. By assuming normally incident plane-wave illumination, from the normalized 2D scattering pattern shown in Fig. 5f, we observe a scattered power peaked at broadside. By changing the uniform phase distribution to a chessboard-like pattern as shown in Fig. 5b^18,41^, the backscattered power is strongly reduced, but there are still strong scattering peaks at other directions, as it can be observed in Fig. 5g. We now apply to the chessboard-like pattern a space-coding matrix varying with the time sequence “10” in a modulation period, as illustrated in Fig. 5c. In this case, the incident power is redistributed in both space and frequency domains. The scattering patterns pertaining to the center frequency and the first five positive harmonic frequencies are displayed in Fig. 5h. Note that this time-coding results in the generation of odd harmonics only, as shown in Supplementary Figure 8a. We clearly observe that the beam shapes are the same as those in Fig. 5g, but the backscattered powers spread to odd sidebands only. This active coding metasurface modulated by the space-time-coding matrix in Fig. 5c could yield zero backscattered power at the central frequency. Moreover, the maximum of backscattered powers at different harmonic frequencies is also reduced by ~9.55 dB by comparison with Fig. 5f, and by ~3.92 dB by comparison with the chessboard-like space coding in Fig. 5g.

If we now consider the BPSO-optimized space-coding shown in Fig. 5d, the backscattered power would be distributed uniformly in all possible directions^18,19,28^, as it can be observed in Fig. 5i. This optimized space-coding results in a more uniform scattering pattern, and decreases the maximum of backscattered power of 7.07 dB by comparison with the chessboard-like space-coding. On this basis, we apply a random time-coding sequence “10011010” to the optimized space-coding, as displayed in Fig. 5e. In this case, the incident power is redistributed in both space and frequency domains more uniformly. The scattering patterns pertaining to the first five positive harmonic frequencies are displayed in Fig. 5j. The beam shapes are also the same as those in Fig. 5i, but the backscattered powers are now significantly reduced in the whole upper space, and spread to almost all sidebands (see Supplementary Figure 8b). This space-time-coding matrix could also yield zero backscattered power at the central frequency. Moreover, the maximum of backscattered energies at different harmonic frequencies are reduced by ~21.52 dB by comparison with Fig. 5f, and by ~8.82 dB by comparison with Fig. 5i. Generally, the backscattered powers can be perfectly suppressed in both space and frequency domains, by introducing proper time modulation on space-coding metasurfaces, which ensures the effective and robust RCS reduction.

### Experimental verification

**Fig. 6:**
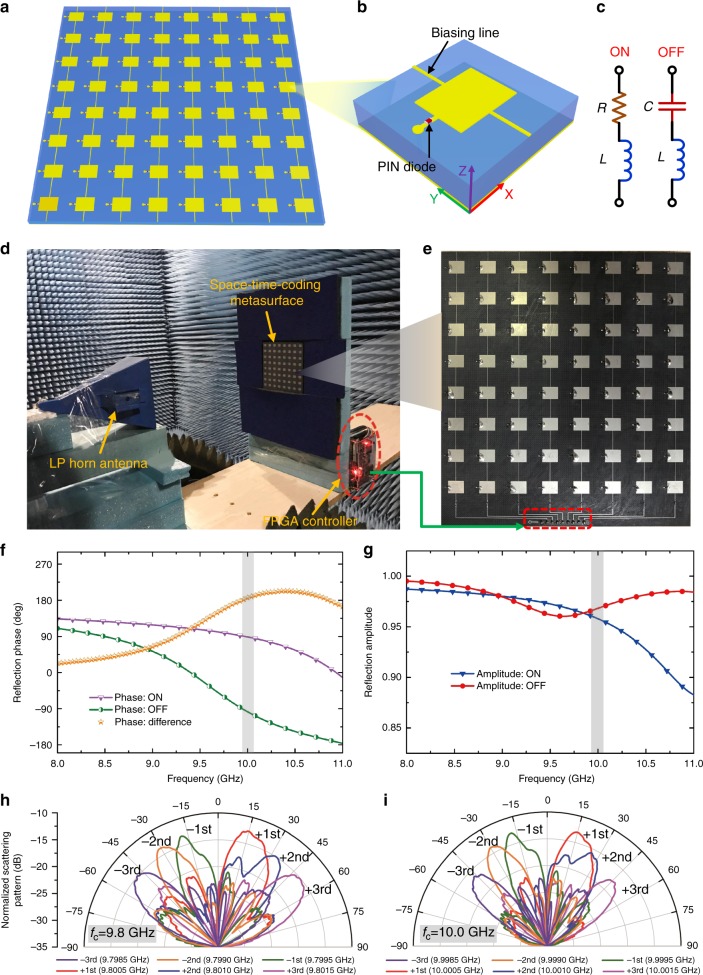
Prototype design, modeling, and characterization. **a** Schematic of the realized space-time-coding metasurface prototype. **b** Geometry of the coding element with the biasing line. **c** Equivalent circuit models of the PIN diode biased at the “ON” and “OFF” states. **d** Measurement setup in an anechoic chamber. **e** Photo of the fabricated prototype. **f**, **g** Numerically computed reflection phase and amplitude of the coding element, respectively, for both diode states as a function of frequency. The grey-shaded areas indicate a neighborhood of the operational frequency (10 GHz). **h**, **i** Measured scattering patterns at different harmonic frequencies, for excitation frequencies of 9.8 and 10 GHz, respectively

To validate the above-illustrated concepts and designs, we realize a coding metasurface composed of 8 × 8 elements pertaining to the harmonic-beam-steering example (see Fig. 3). As shown in Fig. 6a, each column (composed of eight coding elements) is connected by biasing lines with a width of 0.2 mm and shares a common control voltage. Each coding element consists of a rectangular metal patch printed on a grounded F4B substrate with dielectric constant of 2.65, loss tangent of 0.001, and thickness of 2 mm. A PIN-diode (M/A-COM MADP-000907–14020x) is employed to connect the patch with ground through a metal stub and via^42^, as shown in Fig. 6b. The size of the coding element is 15 mm × 15 mm, which corresponds to half wavelength at the central frequency of 10 GHz. The dimensions of the stub and patch are determined to attain a 180° reflection-phase change when the PIN diode switches between “ON” and “OFF” states (see Supplementary Figure 9 for more details). As the diode is “ON” (or “OFF”) with a biasing voltage of 1.33 V (or 0 V), the corresponding coding state is “1” (or “0”). The equivalent circuit model of the PIN diode is illustrated in Fig. 6c, with *R* = 7.8 Ω, *C* = 28 pF, and *L* = 30 pH for full-wave analysis around 10 GHz. Full-wave simulation results are obtained by using the commercial software package, CST Microwave Studio 2014 (https://www.cst.com/products/cstmws). In the simulations of the coding elements, periodic boundaries are applied to the *x* and *y* directions, and two Floquet ports are used along the ±*z* directions. A linearly polarized plane-wave illumination (with *x*-directed electric field) is assumed to calculate the reflection coefficient pertaining to the coding elements. The phase and amplitude of the reflection coefficient pertaining to the “ON” and “OFF” states of the PIN diode are shown in Fig. 6f, g, respectively. We observe that the phase difference is 180° around the frequency of 10 GHz, and the corresponding amplitude is nearly unit (above 0.95).

Based on this design, we fabricate a prototype of the proposed coding metasurface, as shown in Fig. 6e. The structure consists of eight columns with eight connected elements, and has an overall size of 120 mm × 120 mm (4*λ*_c_ × 4*λ*_c_). The experiments are carried out in a standard microwave anechoic chamber, with the measurement setup illustrated in Fig. 6d. A linearly polarized horn antenna working from 8 to 12 GHz serves as the excitation source and is connected to a microwave signal generator (Keysight E8267D), which provides the excitation signal at a fixed frequency. Both the space-time-coding digital metasurface and feeding antenna are mounted on a turntable, which can automatically rotate by 360° in the horizontal plane. Another horn antenna is used to receive the harmonic scattered signals via a spectrum analyzer (Keysight E4447A). An FPGA hardware control board (ALTERA Cyclone IV) is exploited to provide dynamic biasing voltages for the coding metasurface, in which each column shares a control voltage. The adopted FPGA is a low-cost system with a clock speed of 50 MHz, in which a code is preloaded to generate eight control voltages according to the time-coding sequences in Fig. 3a. The modulation period *T*_0_ of the time-coding sequences is 2 μs and the pulse width *τ* is 0.2 μs, which correspond to a system modulation frequency *f*_0_ = 0.5 MHz, and diode switching speed of 5 MHz, respectively. It is worth pointing out that the relatively fast switching speed is attributable to the parallelized design of PIN-diodes in each column in Fig. 6a, e.

Figure 6h, i shows the measured 2D scattering patterns pertaining to the first three positive and negative harmonics, assuming center frequencies of 9.8 and 10.0 GHz, respectively. Due to the blockage effect of the illuminating horn antenna, the scattering patterns at the center frequencies are not shown. The scattering patterns are normalized with respect to the scattered peak power measured from a copper plate with the same size as the metasurface. We clearly observe that the harmonic beam steering is obtained, as an effect of the space-time-coding metasurface. The steering angles of the harmonic beams are in good agreement with the theoretical predictions (see Supplementary Table 1 for more details), thereby validating the effectiveness of the proposed approach. We also observe that the results at the central frequency of 10.0 GHz are moderately worse than those at 9.8 GHz, which is likely attributable to a shift of the 180° phase-difference condition due to fabrication tolerances as well as the various approximations and parameter uncertainties in our modeling. Nevertheless, the measured results at 10.0 GHz still exhibit the general hallmarks of the harmonic beam steering, as shown in Fig. 6i. The proposed space-time-coding metasurface can in principle operate as a broadband system, provided that the design of the coding elements is likewise broadband, which is promising for multi-input and multi-output (MIMO) wireless communications. For instance, at different directions, the scattered power spectra are different for the various harmonic frequencies, and can be precisely controlled. Therefore, we can envision the possibility to encode information in the frequency spectra for multi-point communications, which can find intriguing applications in MIMO scenarios.

## Discussion

In summary, we have proposed a strategy based on time-modulation to design space-time-coding digital metasurfaces, in which each coding element has a set of time-coding sequences switched cyclically in a modulation period. Together with spatial modulation, the proposed time modulation on digital coding can control both spatial (propagation direction) and spectral (frequency distribution) characteristics of the scattered EM power. As a proof of principle, by means of a BPSO-based design of time-coding sequence, we demonstrated beam-scanning patterns at different harmonic frequencies. An FPGA-controlled prototype was fabricated and experimental characterized to validate the proposed method, indicating good agreement with the theoretical predictions. We also proposed other intriguing application scenarios to beam shaping and reduction of the scattered power, via proper design of the 3D space-time-coding matrix.

By comparison with the “phase-switched screens”^34^, our approach is considerably more general and flexible, owing to the judicious combination between the wave-manipulation capabilities enabled by metasurfaces and suitable temporal modulation. This enables more precise and effective control of the scattering and radiation, for both even/odd harmonics and central frequency.

In general, our proposed theory and design of space-time-coding digital metasurfaces substantially expand the application scope of digital metamaterials, and may find important applications to wireless communications, cognitive radars, MIMO systems, OAM beam generation, adaptive beamforming, and holographic imaging. The proposed concept can be extended to the terahertz and optical ranges, as well as to acoustic waves, and can be further generalized to control the transmitted waves by suitably modulating the phase or amplitude of the transmission coefficient. Planned future investigations also include the exploration of space-time coding to engineer nonreciprocal effects.

### Code availability

The custom computer codes utilized during the current study are available from the corresponding authors on reasonable request.

## Electronic supplementary material

Supplementary information

## Data Availability

The datasets generated during and/or analyzed during the current study are available from the corresponding authors on reasonable request.
