# Supplementary material for: Space-time-coding digital metasurfaces
Source: Nat Commun. 2018 Oct 18;9:4334. doi: 10.1038/s41467-018-06802-0 (PMC6194064; doi:10.1038/s41467-018-06802-0)
Supplement: Supplementary file 1 — Supplementary information [file 41467_2018_6802_MOESM1_ESM.pdf]

Supplementary Information for

Space-time-coding digital metasurfaces

by Zhang et al.

Supplementary Note 1: Derivation of Fourier-series coefficients a_{pq}^{m}

The Fourier-series coefficients a_{pq}^{m} of the periodic function $\Gamma_{pq}(t)$ are derived in detail as follows:

$$
\begin{aligned}
a_{pq}^{m} &= \sum_{n=1}^{L} \Gamma_{pq}^{n} c_{pq}^{mn} = \sum_{n=1}^{L} \frac{\Gamma_{pq}^{n}}{T_0} \int_{(n-1)\tau}^{n\tau} \exp\left(-j2\pi m f_0 t\right) dt \\
&= \sum_{n=1}^{L} \Gamma_{pq}^{n} \int_{(n-1)\tau/T_0}^{n\tau/T_0} \exp\left(-j2\pi m t/T_0\right) d\frac{t}{T_0} = \sum_{n=1}^{L} \Gamma_{pq}^{n} \int_{(n-1)/L}^{n/L} \exp\left(-j2\pi m t\right) dt \\
&= \sum_{n=1}^{L} \Gamma_{pq}^{n} \left.\frac{\exp\left(-j2\pi m t\right)}{-j2\pi m}\right|_{(n-1)/L}^{n/L} = \sum_{n=1}^{L} \Gamma_{pq}^{n} \frac{1}{-j2\pi m}\left[\exp\left(-j2\pi m \frac{n}{L}\right) - \exp\left(-j2\pi m \frac{n-1}{L}\right)\right] \\
&= \sum_{n=1}^{L} \frac{\Gamma_{pq}^{n}}{-j2\pi m} \exp\left[-j2\pi m\left(\frac{2n-1}{L}\right)\right]\left[-2j\sin\left(\frac{\pi m}{L}\right)\right] \\
&= \sum_{n=1}^{L} \frac{\Gamma_{pq}^{n}}{L} \operatorname{sinc}\left(\frac{\pi m}{L}\right) \exp\left[\frac{-j\pi m(2n-1)}{L}\right]
\end{aligned}
\tag{1}
$$

The equivalent amplitude A_{pq}^{m} and phase φ_{pq}^{m} excitations of each coding element at different harmonic frequencies can be written as

$$
\begin{aligned}
A_{pq}^{m} &= \left| \sum_{n=1}^{L} \frac{\Gamma_{pq}^{n}}{L} \operatorname{sinc}\left(\frac{\pi m}{L}\right) \exp\left[\frac{-j\pi m(2n-1)}{L}\right] \right| \\
\varphi_{pq}^{m} &= \arg\left\{ \sum_{n=1}^{L} \frac{\Gamma_{pq}^{n}}{L} \operatorname{sinc}\left(\frac{\pi m}{L}\right) \exp\left[\frac{-j\pi m(2n-1)}{L}\right] \right\}
\end{aligned}
\tag{2}
$$

Via Supplementary Equation (2), we can calculate the equivalent amplitude and phase of each coding element for any time-coding sequences, which can help controlling the scattered power at various harmonic frequencies.

Supplementary Note 2: Detailed analysis of the random space-time-coding matrix

The three-dimensional (3D) space-time-coding matrix in Supplementary Figure 1a can be displayed in another form, as shown in Supplementary Figure 1f. Each coding element has its own time-coding sequences, which can be independently designed.

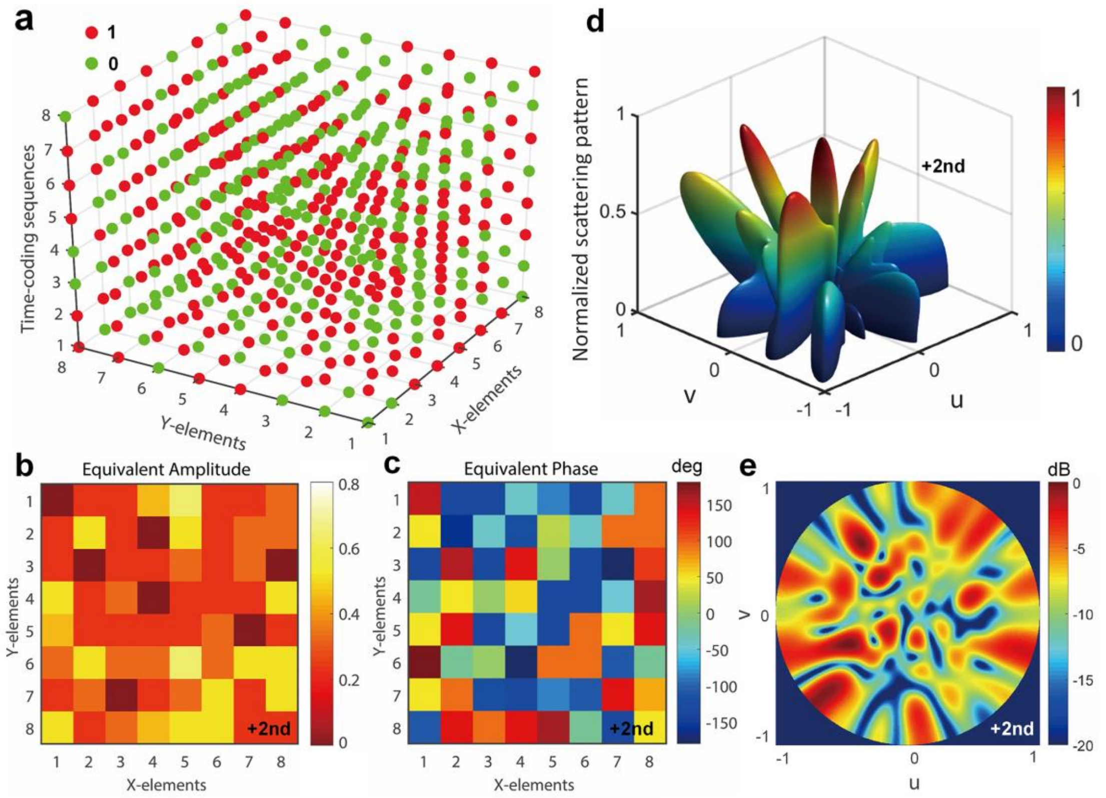

f

X-elements

Y-elements	1	2	3	4	5	6	7	8
1	01101001	01101101	11000010	11011101	11001100	10101110	10011110	00110101
2	01011011	01100100	10011110	10100000	00011001	11111101	10110010	10100011
3	01010100	11100001	11111110	00100000	01011001	01001111	00100100	11000011
4	10011101	10111111	00011000	10101111	11111110	01010100	11010000	01100111
5	10110001	11100011	10100100	11010000	00101100	10110010	11010010	00100000
6	01000111	11011001	10111000	00100100	00110011	00101011	11000100	10011000
7	11110001	00111111	10100000	11101111	00001100	11101100	11100011	00010011
8	11001110	01010111	00101011	01110111	01110110	10001001	10101110	11010011

Supplementary Figure 1 | Space-time-coding matrix and corresponding scattering patterns. a, Random 3D space-time-coding matrix with dimension (8, 8, 8). The red and green dots represent "1" and "0" digits, respectively. **b,c**, Equivalent amplitudes and phases, respectively, of all coding elements at the +2nd harmonic frequency. **d,e**, Corresponding 3D and 2D scattering patterns, respectively, at the +2nd harmonic frequency. **f**, Time-coding sequences of each coding element in panel **a**.

By using Supplementary Equation (2), the equivalent amplitude and phase at any harmonic frequencies are calculated. Supplementary Figures 2a and 2b show the equivalent amplitude and phase patterns from the -3rd to +4th harmonic frequencies based on the space-time-coding matrix in Supplementary Figure 1a. It can be observed that each harmonic frequency has independent distributions of amplitude and phase. However, the phase at the central frequency is only 0 or 180°, which is

inherently limited by the 1-bit design of the coding element. Supplementary Note 3 illustrates how to circumvent this limitation. Further results pertaining to scattering patterns at different harmonic frequencies are shown in Supplementary Figure 3. The scattering patterns at any harmonic frequencies can be obtained via Eq. (7). Due to the different equivalent amplitude and phase distributions at different harmonic frequencies, the resulting scattering patterns are also different.

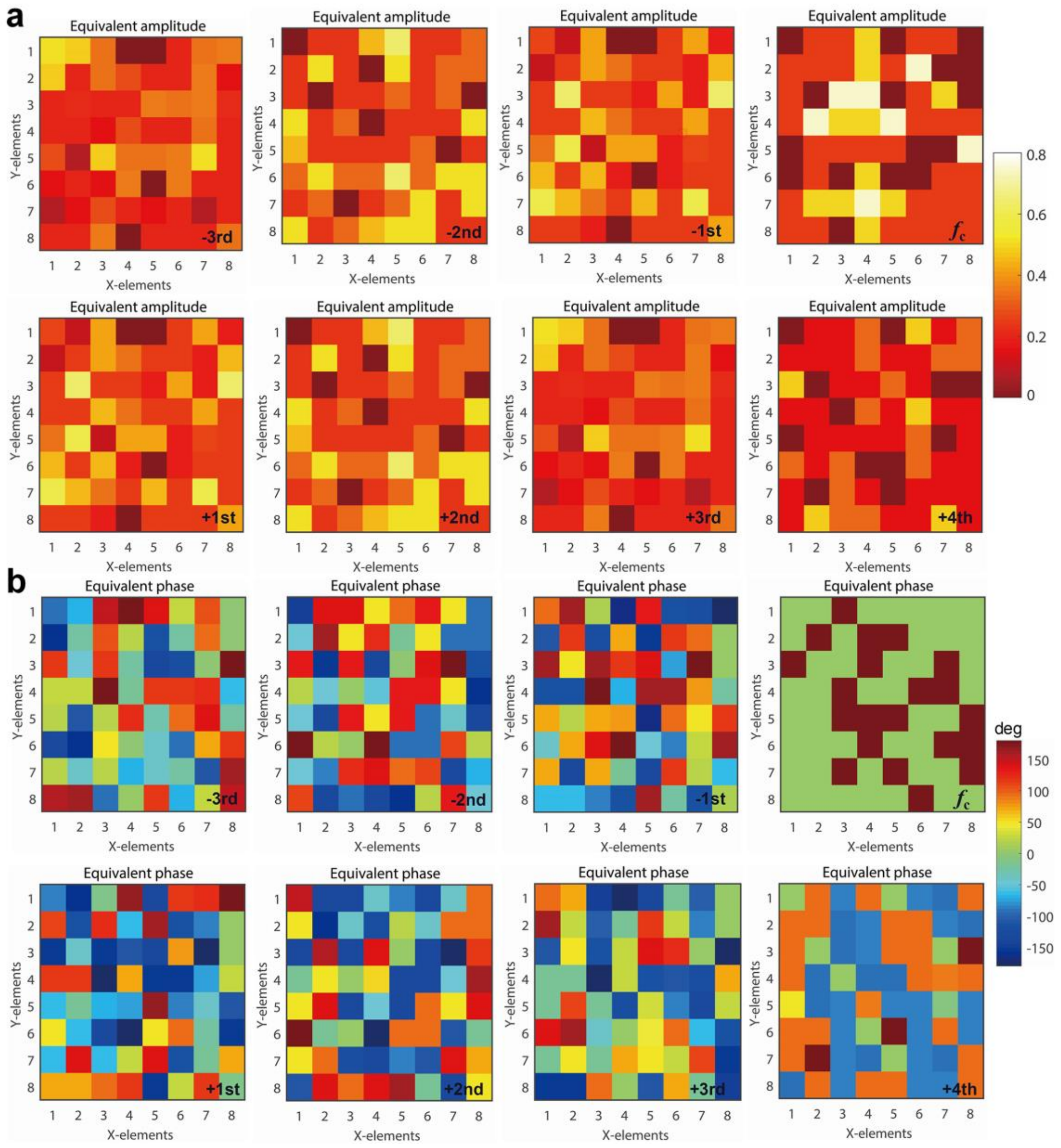

Supplementary Figure 2 | Equivalent amplitudes and phases at any harmonic frequencies. a,b, Equivalent amplitude and phase distributions, respectively, pertaining to the -3rd to +4th harmonic frequencies, based on the space-time-coding matrix in Supplementary Figure 1a.

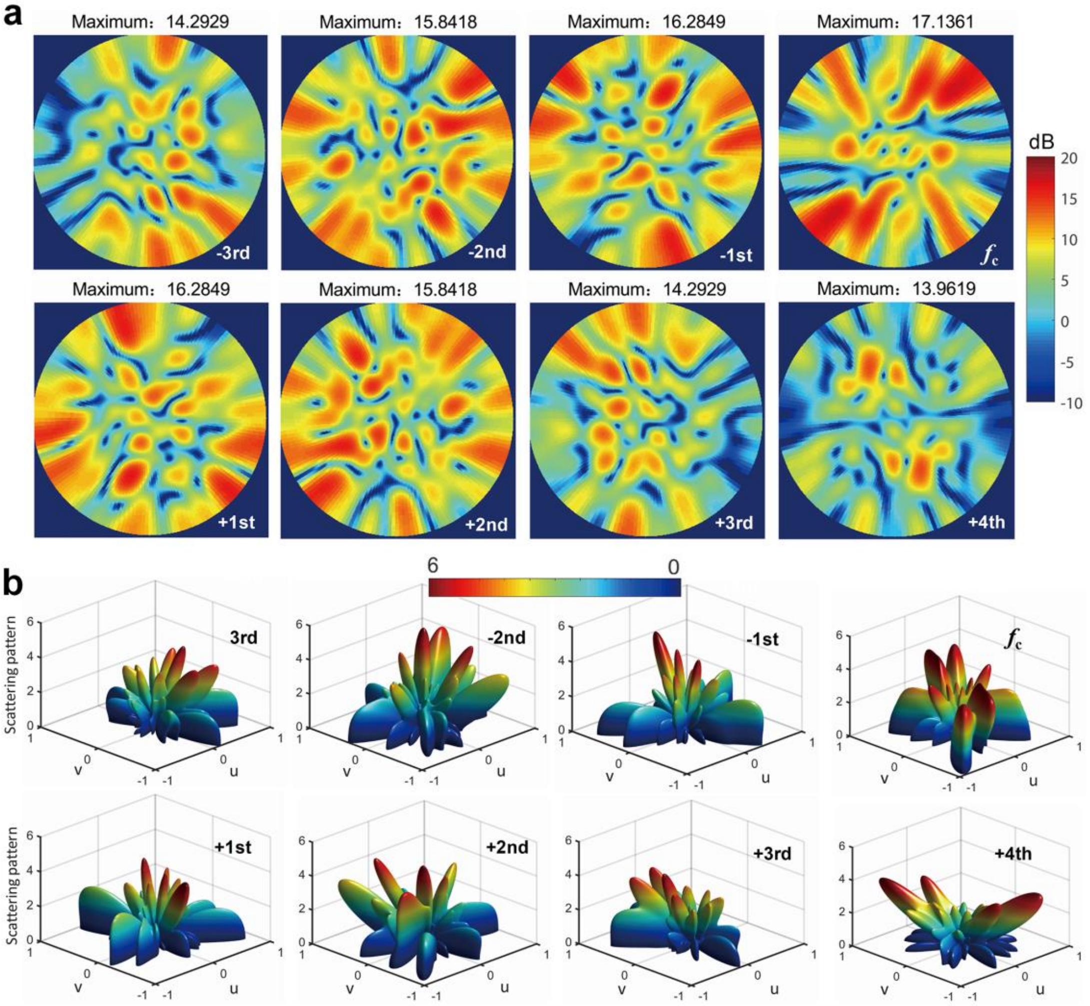

Supplementary Figure 3 | Scattering patterns at different harmonic frequencies. a,b, 2D and 3D scattering patterns, respectively, pertaining to the -3rd to +4th harmonic frequencies based on the space-time-coding matrix in Supplementary Figure 1a.

Supplementary Note 3: Equivalent phase coverages at different harmonic frequencies

As previously mentioned, the equivalent phases φ_{pq}^{m} of each coding element at any harmonic frequencies can be independently designed via suitable choice of the corresponding time-coding sequence. Here, we theoretically study the phase coverage at different harmonic frequencies. In the 1-bit case, the coding elements only exhibit 0 or 180° phase responses, and the equivalent phase coverages are demonstrated in Supplementary Figure 4a. We can observe that the phase coverage can reach 360° at any harmonic frequencies, with the exception of the central frequency. This can be

explained by recalling that the equivalent phase φ_{pq}^{0} at the central frequency is given by

$$\varphi_{pq}^{0} = \arg\left(\sum_{n=1}^{L} \frac{\Gamma_{pq}^{n}}{L} \right) \tag{3}$$

which clearly indicates that the value of φ_{pq}^{0} is either 0 or 180°. To relax this restriction, we further study the phase coverages of 2-bit digital coding elements, which have four phase states as 0, 90°, 180°, and 270°. As shown in Supplementary Figure 4b, the equivalent phase coverages at the central frequency can also reach almost 360°, provided that the time-coding sequences of each coding elements are sufficiently long.

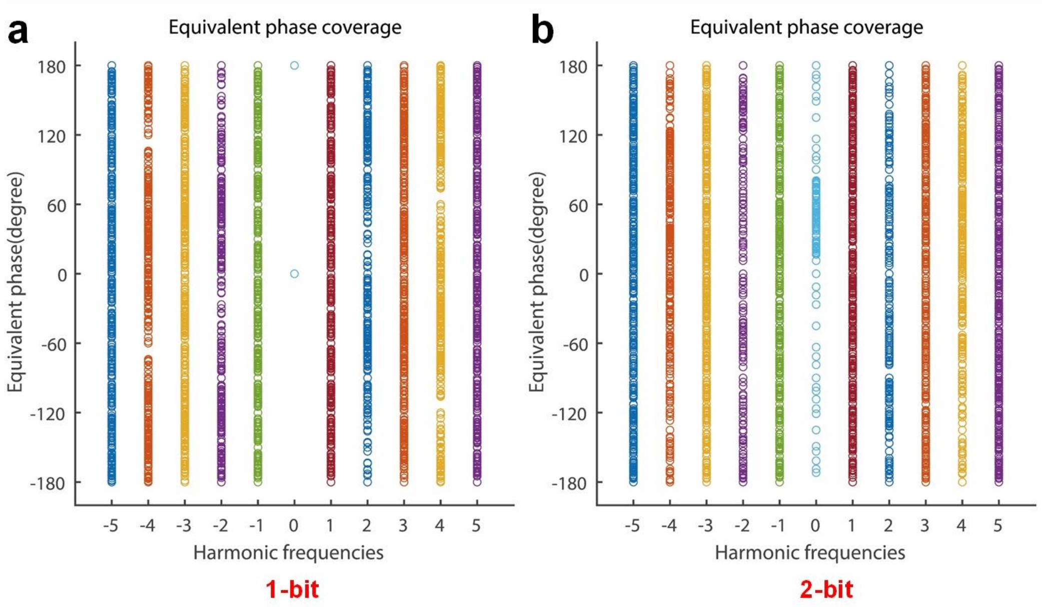

Supplementary Figure 4 | a,b, Equivalent phase coverages of 1-bit and 2-bit digital coding elements, respectively, at different harmonic frequencies.

On the other hand, the control system and biasing circuit of conventional (n>2)-bit programmable metasurfaces are very complicated. In order to design non-modulated elements with higher-bit coding, such as 3-bit, additional diodes need to be embedded into the elements. The layout would be very complicate and the control system would also be a challenge. However, the proposed space-time-coding metasurfaces provide a simple strategy to obtain almost 360° phase coverage at any harmonic frequencies and

central frequency. Therefore, high-bit programmable metasurfaces can be easily realized by suitably designing the time-coding sequences, which does not need a complicate layout and control system.

Supplementary Note 4: Alternative explanation of the harmonic beam steering in Figure 2

For a better understanding of our work, we provide an alternative explanation of the harmonic beam steering, by introducing the time shift in the Fourier transform. As shown in Figures 2a and 2b, the time-coding sequences of the Y-elements from 1st to 8th can be considered as the periodic function $\Gamma_{pq}(t)$ with a time shift t_q,

$$\Gamma_{pq}(t-t_q) \xleftrightarrow{\mathcal{FS}} a_{pq}^{m} \exp\left(-j2\pi m f_0 t_q\right) \tag{4}$$

where $t_q = (q-1)\tau = (q-1)T_0/8$, and the Fourier series coefficients a_{pq}^{m} of the periodic function $\Gamma_{pq}(t)$ are given in Eq. (6). When the time shift t_q is applied to the periodic function $\Gamma_{pq}(t)$, there will be an additional space phase shift $-2\pi m f_0 t_q$ with unchanged amplitude at the *m*th harmonic frequency, which clearly explains the phase gradients in Figures 2c and 2d. Therefore, the phase difference $\vartriangle\psi_m$ between adjacent $(p, q)^{th}$ and $(p, q+1)^{th}$ elements at the *m*th harmonic frequency can be written as

$$\vartriangle\psi_m = -2\pi m f_0 (t_q - t_{q+1}) = m\pi/4 \tag{5}$$

Under normal incidence, the generalized Snell's law[2] can be simply expressed as

$$k \sin\theta_m = \frac{\vartriangle\psi_m}{d_y} \tag{6}$$

where θ_m is the beam steering angle at the *m*th harmonic frequency $f_c + m f_0$, $k = 2\pi/\lambda_c$ is the wavenumber of the reflected waves, and $d_y = \lambda_c/2$ is the element period along the *y* direction. Therefore, the beam steering angle θ_m can be written as:

$$\theta_m = \arcsin\left(\frac{\lambda_c}{2\pi}\frac{\Delta\psi_m}{d_y}\right) = \arcsin(m/4) \quad (7)$$

Finally, we calculate the steering angles at different harmonic frequencies, $\theta_0 = 0^\circ$, $\theta_{\pm1} = \pm 14.48^\circ$, $\theta_{\pm2} = \pm 30^\circ$ and $\theta_{\pm3} = \pm 48.59^\circ$, which are all in good agreement with the theoretical results in Figures 2e and 2f.

Supplementary Note 5: Details on the BPSO-based design for harmonic beam steering

As discussed in the main text, we design a coding metasurface with 8×8 elements and a time-modulation period with 10 intervals. For simplicity, each column of eight coding elements shares the same control voltage, which corresponds to the same digital code. In this case, the 3D space-time coding matrix is simplified to a 2D matrix (similar to the process that associates Figure 2a to Figure 2b). In the PSO algorithm, [1,2] a potential solution is regarded as a particle. Every particle is represented as an *n*-dimensional vector, and every element of the vector is described by the digits '0' or '1', corresponding to a bit of the space-time coding matrix. The moving velocity of the particle indicates the probability of position change, and is given by

$$v_{\mathrm{ij}}^{k+1} = w \cdot v_{\mathrm{ij}}^{k} + c_1 \cdot \mathrm{rand}_1() \cdot (\mathrm{pbest}_{ij}^{k} - x_{ij}^{k}) + c_2 \cdot \mathrm{rand}_2() \cdot (\mathrm{gbest}_{ij}^{k} - x_{ij}^{k}) \quad (8)$$

where $\mathrm{rand}_1()$ and $\mathrm{rand}_2()$ are random numbers between 0 and 1, c_1 and c_2 are the acceleration constants, w is the inertia coefficient, pbest_{ij}^{k} is the individual optimal position, and gbest_{ij}^{k} is the global optimal position. Consistently with the probabilistic interpretation, the velocity v_{ij} is constrained within the interval [0, 1], by means of a sigmoid-function mapping:

$$S(v_{ij}) = \mathrm{Sigmoid}(v_{ij}) = \frac{1}{1+\exp\left(-v_{ij}\right)} \quad (9)$$

Accordingly, the position x_{ij}^{k+1} is updated via

$$x_{\mathrm{ij}}^{k+1}=\begin{cases}1, & \mathrm{rand}<S(v_{\mathrm{ij}}^{k+1})\\ 0, & \mathrm{rand}\geq S(v_{\mathrm{ij}}^{k+1})\end{cases} \tag{10}$$

The targets to be optimized are then evaluated by the function $fitness(x)$, looking for the best space-time-coding matrix. In order to achieve the beam steering of the desired angles at the various harmonic frequencies, the fitness function is defined as

$$\mathrm{fitness}(x)=w_1\cdot\left(\sum_{m=-3}^{3}\left|\theta_0^m(x)-\theta_{\mathrm{d}}^m\right|\right)+w_2\cdot\left(\mathrm{SLL}_{\max}^{0,\pm1,\pm2,\pm3}(x)-\mathrm{SLL}_{\mathrm{d}}\right) \tag{11}$$

where θ_0^m and θ_{d}^m denote the simulated and desired steering angles, $\mathrm{SBL}_{\max}^{0,\pm1,\pm2,\pm3}$ and $\mathrm{SBL}_{\mathrm{d}}$ represent the maximum values of the simulated and desired sidelobe levels, and w_1 and w_2 are the corresponding weighting factors of each term, which are set as 5 and 1 in our examples.

Supplementary Note 6: Equivalent amplitudes and phases for the space-time-coding matrix in Figure 3a

We have realized harmonic beam steering based on the phase modulation with the BPSO-based design of the coding matrix shown in Figure 3a. Here, we illustrate the equivalent amplitudes and phases of the coding elements at different harmonic frequencies, as shown in Supplementary Figure 5. At the central frequency, the phase of the 5th coding element is 180°, and different from other elements, but its amplitude is zero. Therefore, the main beam still points at broadside, which is consistent with the results shown in Figures 3b and 3d. Furthermore, the equivalent phases at positive and negative harmonic frequencies also exhibit different phase gradients.

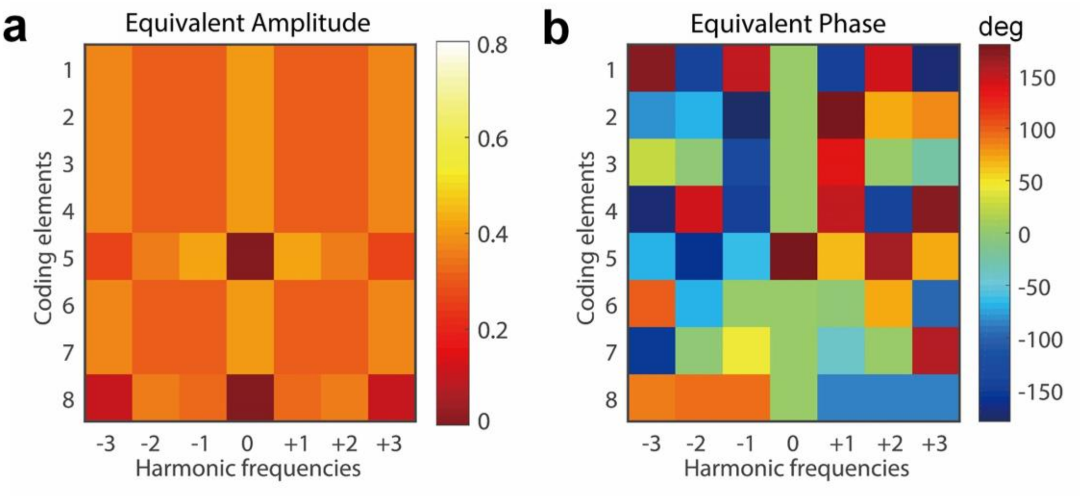

Supplementary Figure 5 | a,b, Equivalent amplitude and phase distributions, respectively, for the BPSO-based design of the space-time coding matrix in Figure 3a.

Supplementary Note 7: Criterion for selecting the time-coding sequences for obtaining 3-bit equivalent coding responses

In some practical applications, sideband signals are not desirable and should be suppressed to improve the scattered/radiated power at the central frequency. As an application for the beam steering shown in Figures 4e-4g, the criterion for selecting time-coding sequences of the eight elements is to make the equivalent amplitudes small enough at all harmonic frequencies, while making the equivalent phases at the central frequency to exhibit 3-bit coding responses with high amplitude (as shown in Figures 4c and 4d). Hence, an in-house MATLAB code based on Supplementary Equation (2) is utilized to search for the eligible time-coding sequences (as shown in Figure 4b).

As a result, the 2D scattering patterns pertaining to the -3rd to +3th harmonic frequencies based on the space-time-coding matrix in Figure 4a are shown in Supplementary Figure 6a. The corresponding 1D scattering patterns at the first five negative and positive harmonic frequencies are also shown in Supplementary Figure 6b. We observe that the maximum sideband level is about -12.95dB, which is much lower than the pattern level at the central frequency (cf. Figure 5f).

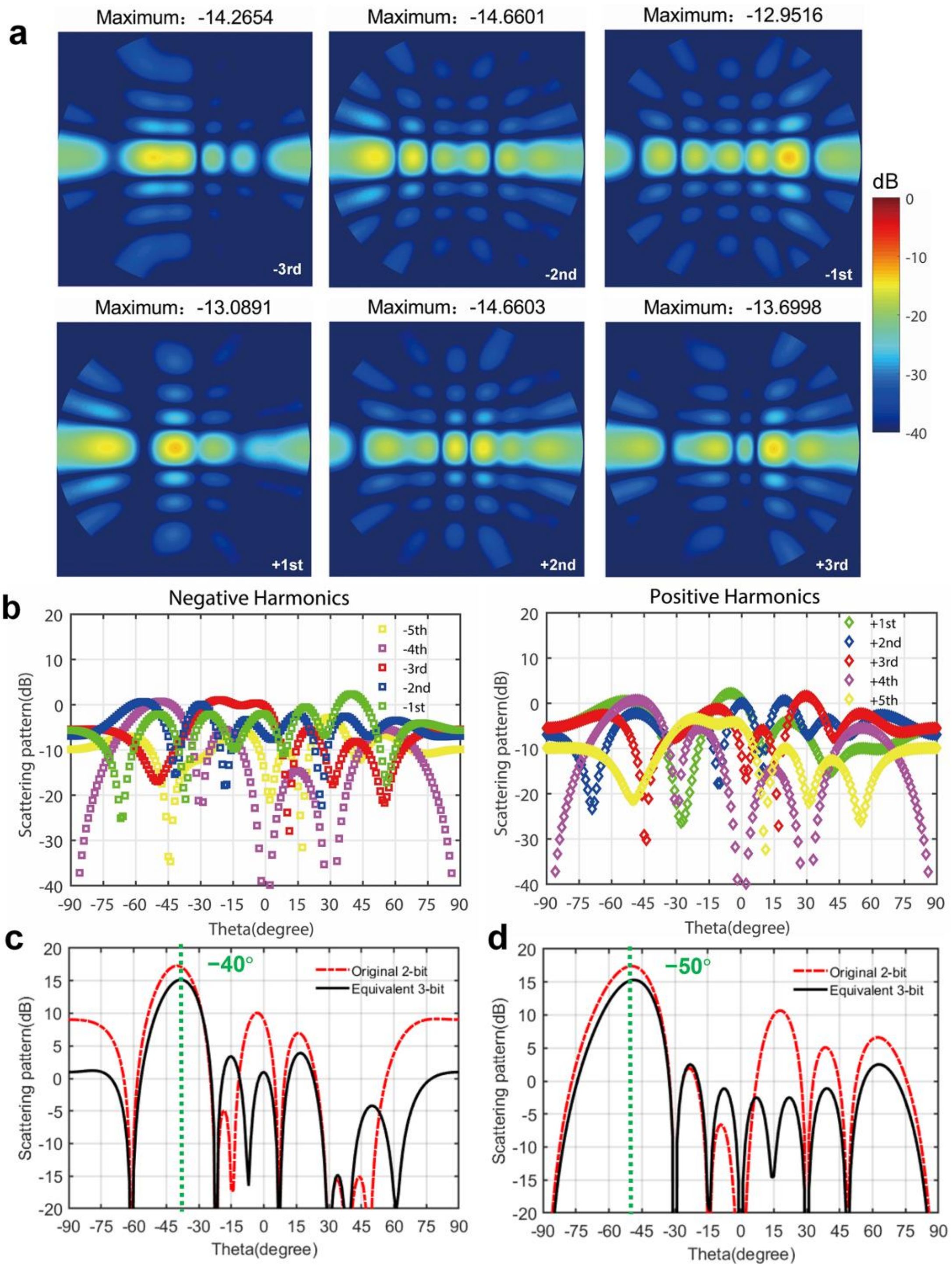

Supplementary Figure 6 | a, 2D scattering patterns pertaining to the -3rd to +3th harmonic frequencies, based on the space-time-coding matrix in Figure 5a. **b**, Corresponding 1D scattering pattern cuts (at $\varphi = 90°$) at different harmonic sidebands. **c,d**, Comparison of scattering patterns pertaining to the original 2-bit coding and equivalent 3-bit coding for realizing larger steering angles at the central frequency.

Moreover, we can also utilize the equivalent 3-bit coding to attain larger steering angles. Supplementary Figure 6c compares the performance of the original 2-bit

coding “3-0-2-3-0-1-3-0” with the equivalent 3-bit coding “ 7′-1′-4′-6′-1′-3′-6′-0′ ”. It is observed that both schemes could realize a steering angle of 40°, but the latter accompanied by a lower sidelobe level. A similar example is shown in Supplementary Figure 6d, in which the original 2-bit coding is “2-0-1-3-0-2-3-1”, and the equivalent 3-bit coding is “ 5′-0′-3′-6′-1′-4′-7′-2′ ” for a steering angle of 50°.

a

X-elements

Y-elements	1	2	3	4	5	6	7	8
1	22221232	23322233	23322233	23322233	32303333	32303333	32303333	32303333
2	22221232	22221232	23322233	23322233	32303333	32303333	32303333	03000333
3	22221232	22221232	22221232	23322233	32303333	32303333	03000333	03000333
4	22221232	22221232	22221232	22221232	32303333	03000333	03000333	03000333
5	21212211	21212211	21212211	11111210	00103000	00103000	00103000	00103000
6	21212211	21212211	11111210	11111210	10110100	00103000	00103000	00103000
7	21212211	11111210	11111210	11111210	10110100	10110100	00103000	00103000
8	11111210	11111210	11111210	11111210	10110100	10110100	10110100	00103000

b

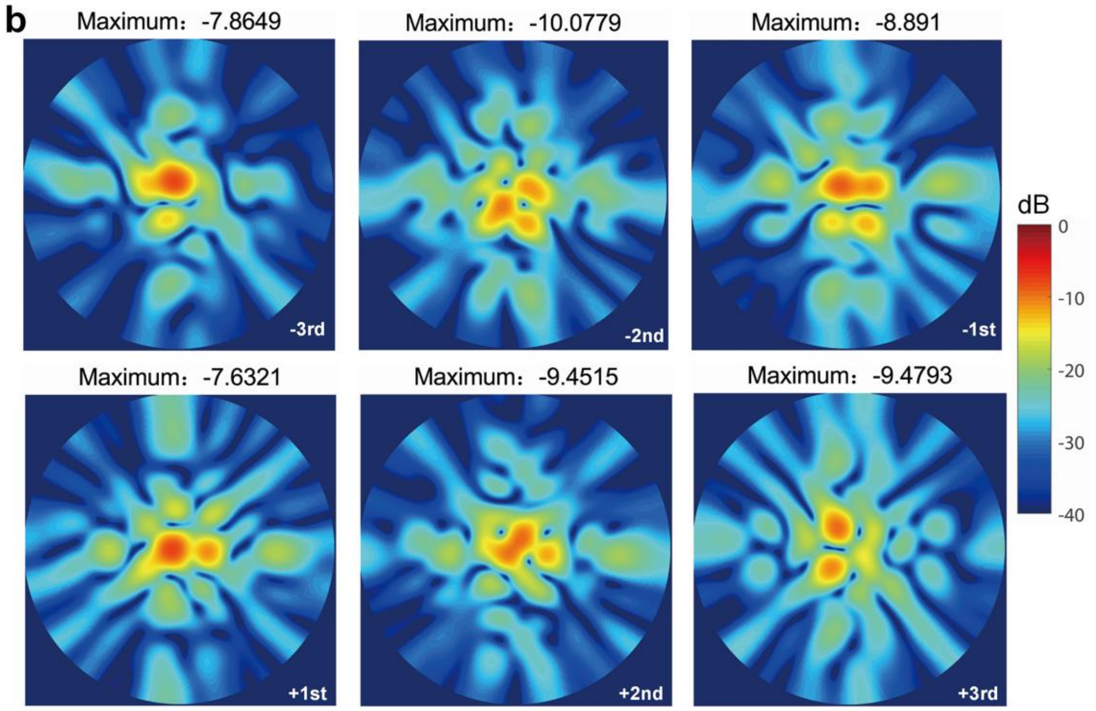

Supplementary Figure 7 | Space-time-coding configuration for vortex-beam generation and corresponding 2D scattering patterns at harmonic frequencies. a, The coding metasurface is divided into eight sectors, each characterized by a 2-bit time-coding sequence. **b**, Corresponding 2D scattering patterns pertaining to the -3rd to +3th harmonic frequencies.

Furthermore, the eight sets of time-coding sequences with rotated distribution (see Supplementary Figure 7a) can be used to generate a vortex-beam at the central frequency, and the scattered powers at the harmonic frequencies (see Supplementary Figure 7b) are also much smaller than that in Figure 4j. Finally, we can also utilize

optimization algorithms to reduce power losses in harmonic sidebands, so as to further improve the efficiency of the coding metasurface.

Supplementary Note 8: Spectral analysis of the maximum intensity of harmonics

Figures 5h and 5j show the spatial scattering patterns at different harmonic frequencies. Here, we investigate the spectral distribution of the maximum intensity of the harmonics. Supplementary Figure 8a and 8b show the maxima of scattering patterns from -50th to +50th harmonic frequencies, corresponding to the coding matrices in Figure 5c and 5e, respectively. It can be observed that the maximum intensity gradually decreases with the increase of the harmonic order, and drops significantly with respect to the first few harmonics.

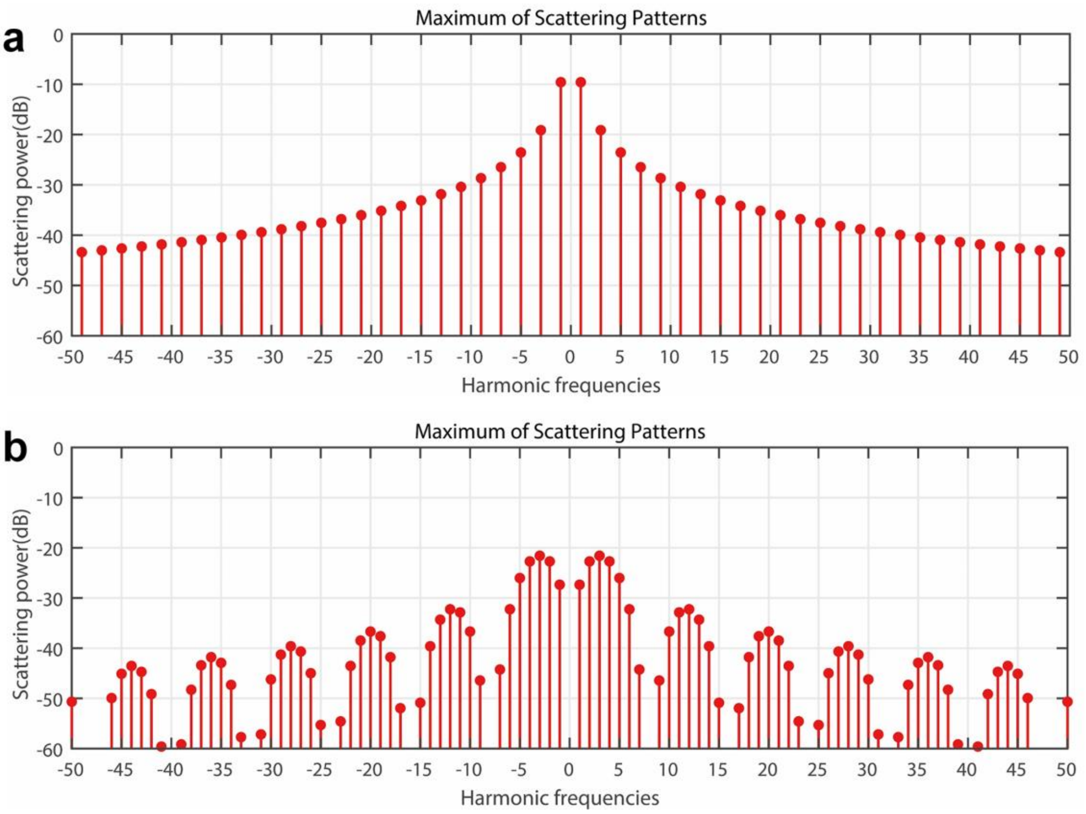

Supplementary Figure 8 | a,b, Maxima of the scattering patterns from -50th to +50th harmonic frequencies, corresponding to the coding matrices in Figures 5c and 5e, respectively.

As we previously mentioned, the space-time-coding in Figure 5e yields better RCS

reduction performance than that in Figure 5c. This mainly depends on the judiciously designed coding sequences in both space- and time-domain, which can redistribute the scattered power more uniformly not only in space but also in spectrum, as demonstrated in Supplementary Figure 8. In connection with the time-domain response of the proposed space-time-coding metasurface, we observe that, by assuming the modulation time much larger than the period at the central wavelength, and neglecting the transients, during each time intervals one effectively experiences a time-harmonic response associated with a specific spatial coding. Especially for the case of RCS reduction in Figure 5e, the time-domain response of this 3D space-time-coding matrix during any modulation interval can be represented by the scattering pattern in Figure 5i. In this case, the scattered power at any direction remains at low levels due to the optimized space-coding in Figure 5d for redistributing the power in the space-domain uniformly.

As also indicated in the main text, the scattering patterns in Figure 5h and 5j are similar in terms of beam shapes, only differing in the maximum value. The scattering patterns at different harmonic frequencies overlap in the space-domain and therefore the temporal interference of different harmonics may add up to a ultrashort high-intensity pulse in the time-domain. Here, we provide two solutions to limit the maximum intensity over time.

(1) We could utilize the random 3D space-time-coding matrix in Supplementary Figure 1a to randomly redistribute scattering patterns in space. In this case, scattering patterns at different harmonic frequencies would have different beam shapes and would not exactly overlap in space, as shown in Supplementary Figure 3. Therefore, the chances for temporal interference of different spectral harmonics adding up to a short, high-intensity pulse would be further minimized.

(2) On the other hand, we could significantly increase the modulation frequency f_0 to obtain a very short period T_0, which mainly depends on the switching speed of the PIN diodes and the control systems. The PIN diodes we used can reach a 2~3

ns switching speed. By decreasing the switching time to a *nanosecond* scale, it would be difficult for a radar detector to harvest energy from many harmonics. This also represents a possible solution to avoid accumulating a high-intensity pulse.

Supplementary Note 9: Detailed analysis of the proposed 1-bit coding element and experimental results

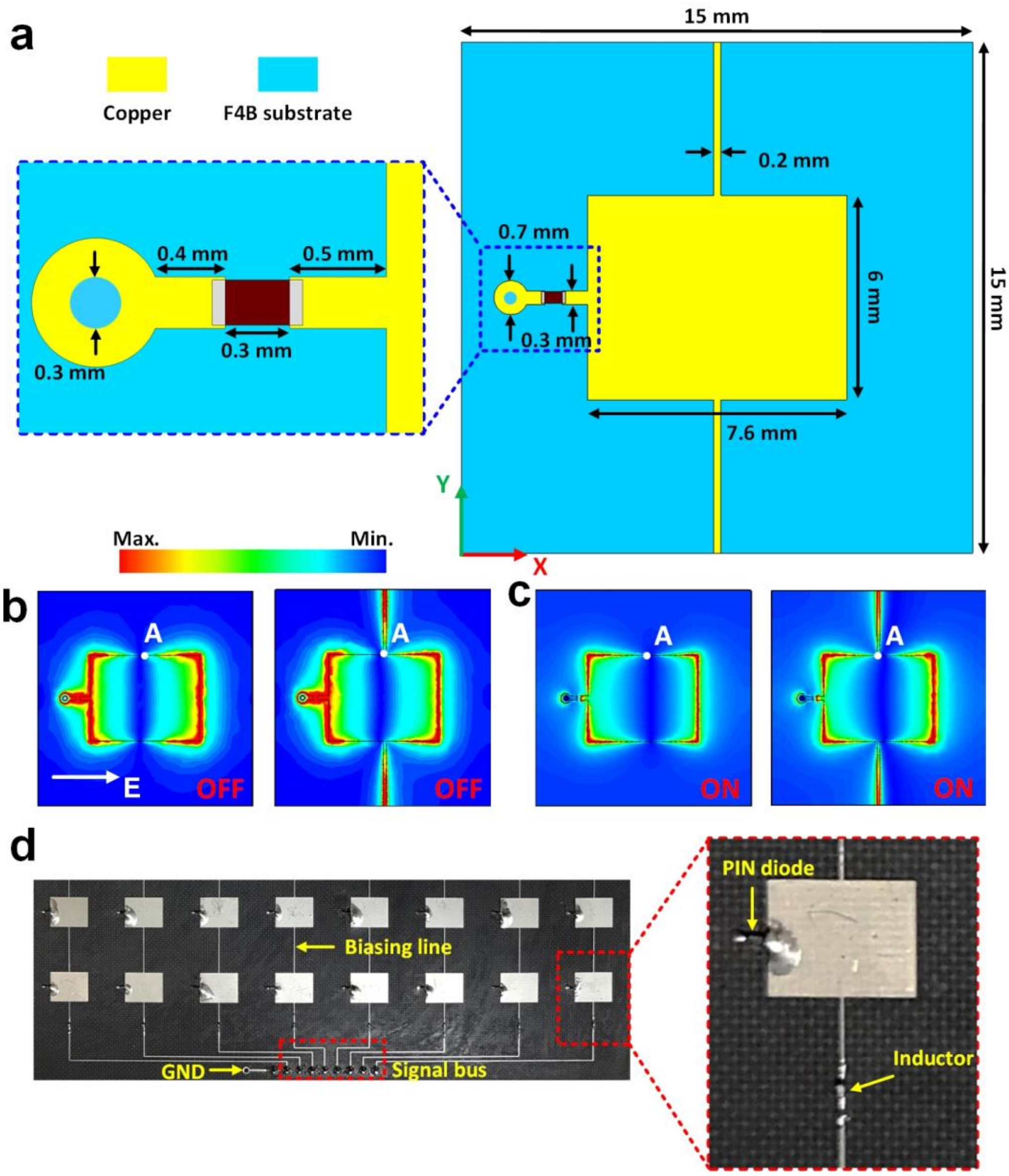

Supplementary Figure 9. a, Top view of the 1-bit coding element with detailed geometrical parameters. **b,c**, Electric-field distributions of the coding element with the PIN diode 'OFF' and 'ON', respectively. **d**, Magnified view of the fabricated prototype in Figure 6b.

The detailed parameters of the coding element considered in our fabricated prototype

are shown in Supplementary Figure 9a. The corresponding electric-field distributions (at the operational frequency of 10 GHz) with and without biasing lines are shown in Supplementary Figures 9b and 9c. When the biasing line is added at point A, it does not affect the field distribution of the rectangular patch at both "ON" and "OFF" states, because the electric field at point A is very weak. Therefore, the introduction of the biasing line does not sensibly affect the performance of the space-time-coding metasurface. Moreover, inductors with inductance of 2nH are also introduced in the biasing lines to provide an RF choke, as illustrated in Supplementary Figure 9d.

Finally, Supplementary Table 1 quantitatively illustrates the comparison between theoretically calculated and measured beam-steering angles at different harmonic frequencies, from which a generally good agreement is observed.

Supplementary Table 1. The comparison of theoretical and measured results.

Harmonic orders	-3rd	-2nd	-1st	+1st	+2nd	+3rd
Theoretically calculated	$-47°$	$-30°$	$-15°$	$15°$	$30°$	$47°$
Measured at 9.8GHz	$-50°$	$-31°$	$-18°$	$16°$	$33°$	$50°$
Measured at 10.0GHz	$-47°$	$-30°$	$-17°$	$15°$	$28°$	$47°$

Supplementary References

1 Zhang, Q. *et al.* Shaping electromagnetic waves using software-automatically-designed metasurfaces. *Sci. Rep.* **7**, 3588 (2017).

2 Nezamabadi-Pour, H., Rostami-Shahrbabaki, M. & Maghfoori-Farsangi, M. Binary particle swarm optimization: challenges and new solutions. *CSI J. Comput. Sci. Eng.* **6**, 21-32 (2008).
